# Cancer and HIV-1 Infection: Patterns of Chronic Antigen Exposure

**DOI:** 10.3389/fimmu.2020.01350

**Published:** 2020-06-30

**Authors:** Selena Vigano, Sara Bobisse, George Coukos, Matthieu Perreau, Alexandre Harari

**Affiliations:** ^1^Ludwig Institute for Cancer Research, University of Lausanne and Department of Oncology, University Hospital of Lausanne, Lausanne, Switzerland; ^2^Service of Immunology and Allergy, University Hospital of Lausanne, Lausanne, Switzerland

**Keywords:** HIV infection, cancer, lymphocytes, cellular immunity, exhaustion, senescence, anergy, immune checkpoint

## Abstract

The main role of the human immune system is to eliminate cells presenting foreign antigens and abnormal patterns, while maintaining self-tolerance. However, when facing highly variable pathogens or antigens very similar to self-antigens, this system can fail in completely eliminating the anomalies, leading to the establishment of chronic pathologies. Prototypical examples of immune system defeat are cancer and Human Immunodeficiency Virus-1 (HIV-1) infection. In both conditions, the immune system is persistently exposed to antigens leading to systemic inflammation, lack of generation of long-term memory and exhaustion of effector cells. This triggers a negative feedback loop where effector cells are unable to resolve the pathology and cannot be replaced due to the lack of a pool of undifferentiated, self-renewing memory T cells. In addition, in an attempt to reduce tissue damage due to chronic inflammation, antigen presenting cells and myeloid components of the immune system activate systemic regulatory and tolerogenic programs. Beside these homologies shared between cancer and HIV-1 infection, the immune system can be shaped differently depending on the type and distribution of the eliciting antigens with ultimate consequences at the phenotypic and functional level of immune exhaustion. T cell differentiation, functionality, cytotoxic potential and proliferation reserve, immune-cell polarization, upregulation of negative regulators (immune checkpoint molecules) are indeed directly linked to the quantitative and qualitative differences in priming and recalling conditions. Better understanding of distinct mechanisms and functional consequences underlying disease-specific immune cell dysfunction will contribute to further improve and personalize immunotherapy. In the present review, we describe relevant players of immune cell exhaustion in cancer and HIV-1 infection, and enumerate the best-defined hallmarks of T cell dysfunction. Moreover, we highlight shared and divergent aspects of T cell exhaustion and T cell activation to the best of current knowledge.

## Introduction

The primary function of the human immune system is to protect the host by reacting upon the encounter of foreign antigens, as well as to prevent autoimmunity through self-recognition. Two arms orchestrate the activation of the immune system: the innate response triggered within the first hours and the adaptive response mounted over the following days, able to recognize and target specific antigens and to generate memory. T cells are the major component of the adaptive immune system consisting of CD4 and CD8 T cells (1), being the latter key players in the physical elimination of tumor and virus-infected cells.

Most naïve T cells encounter their targets, presented by professional antigen presenting cells (i.e., dendritic cells, DCs), in secondary lymphoid organs (2). Such priming is crucial for determining the acquisition of functional attributes by T cells (3, 4). DCs govern the nature of primed T cells *via* the provision of processed antigens in the form of peptide/MHC complexes (signal I) and other important signals, including costimulatory interactions (signal II) and inflammatory cytokines (signal III) (5). Once activated, T cells undergo massive clonal expansion, differentiate into potent effectors, and express chemokines and homing receptors necessary for migration into peripheral tissues. Effector CD4 T cells produce several cytokines depending on the polarization determined by the cognate antigen and the extracellular milieu, effector CD8 T cells express cytotoxic molecules, such as perforin and granzymes, and produce effector cytokines. The production of cytotoxic molecules and cytokines is needed to help contain the spread of pathogens and tumors. The fate of naïve CD8 T cell differentiation is also determined by interdependent variables such as frequency of contact with the immunological synapses, epitope antigenicity, T cell receptor (TCR) affinity for cognate targets and the presence of CD4 T cell help (6). After CD8 T cell expansion and antigen elimination, any further immune activation is prevented by the upregulation and engagement of co-inhibitory molecules such as Cytotoxic T Lymphocyte Antigen-4 (CTLA-4) and Programmed Death-1 (PD-1). Most effector T cells die by apoptosis (contraction phase), but about 5–10% survive and differentiate into memory T cells. Different theories for memory T cell development have been suggested (7), but recent findings strongly suggest that long-lived memory CD8 T cells would arise from a subset of effector T cells through a process of dedifferentiation (8). Memory T cells are then maintained in the absence of antigens (homeostatic expansion) and can exert rapid effector functions in response to previously encountered antigens (1, 9).

Any disturbance of conventional activation signals may drive T lymphocytes to alternative cell fates, i.e., anergy, tolerance and exhaustion. This plasticity has evolved to constrain autoimmunity and excessive immune responses that would otherwise cause undesired tissue damage and immune-pathological conditions. Whereas, anergy is established during priming, due to the absence of costimulatory signals, and senescence is defined as growth arrest after extensive proliferation, exhausted T cells arise from cells which initially gained effector functions but became gradually dysfunctional due to continuous TCR stimulation by persistent antigens (10). Overlapping and discriminating functional and molecular features of these alternative cellular conditions have been comprehensively investigated (11, 12). In the present review, we describe the establishment and hallmarks of T cell exhaustion in HIV-1 infection and cancer. In addition, we highlight the parameters that allow the discrimination between functionally distinct T cell states, which are exhausted, activated, and memory T cells.

## Emergence of T Cell Exhaustion

T cell exhaustion was initially described in the mouse model of LCMV infection (13–16), where, initially functional (17) and then transcriptional analyses led to the identification of PD-1 as first and main molecule associated with this status (15, 18, 19). Afterwards, high PD-1 levels have been observed in Simian Immunodeficiency Virus (SIV) infected Rhesus Macaques (15, 20–22) as well as in HIV-1 infected patients (23–25) and this was related to T cell impaired function and disease progression. In HIV-1 infection, T cell exhaustion is caused by antigen persistency and impaired CD4 T cell help (26, 27). During the acute phase of the infection, CD8 T cell responses are generated, but they are incapable of mediating complete virus clearance. HIV-1 is, indeed, endowed with a high mutation rate capacity that leads to a quick and efficient escape from immune cells (28, 29). Moreover, lymphoid follicles have been shown to be enriched in HIV-1/SIV-infected CD4 cells, and poorly infiltrated by CD8 T cells during early SIV infection. Consistently, the frequency of SIV-specific CD8 T cells entering the lymphoid follicles is inversely associated with the levels of infected cells, suggesting a new mechanism of viral persistency (30). While infected cells are not eradicated, T cells are continuously exposed to viral antigens, leading to a permanent expression of negative receptors and consequently to T cell dysfunction (15, 31–34). Of note, beside antigen escape, HIV-1 preferentially infects HIV-1-specific CD4 T cells (35), leading to profound consequences in the immune-pathogenesis of the disease (28). HIV-1-specific CD4 T cells expand at high frequency during the early phase of the infection. Later on, their number decreases in blood and secondary lymphoid organs (36), due to killing by HIV-1-specific CD8 T cells, virus cytopathic effects and pyroptosis triggered by abortive viral infection (37). In an early stage, CD8 T cell responses are also quickly impaired (27, 38–40), nevertheless this loss of function is partially restored in the presence of HIV-1 specific CD4 T cells (13, 27), highlighting the importance of CD4 T cell depletion in determining CD8 T cell exhaustion. CD4 T cells indeed provide help for CD8 T cells by producing supportive cytokines including interleukin (IL)-2 and IL-21, which can act directly on the responding CD8 T cells (41–48). IL-2 has a pivotal importance during priming of CD8 T cell response, in order to generate functional memory cells able to perform homeostatic turnover and to mount potent secondary responses (49). IL-21 instead has a major role in sustaining and expanding memory CD8 T cells (43, 44). In mice, CD4 T cell help has been recapitulated by CD27 agonism that enhanced specific CD8 T cell effector functions in response to vaccination or a viral infection (50).

**Figure 1:**
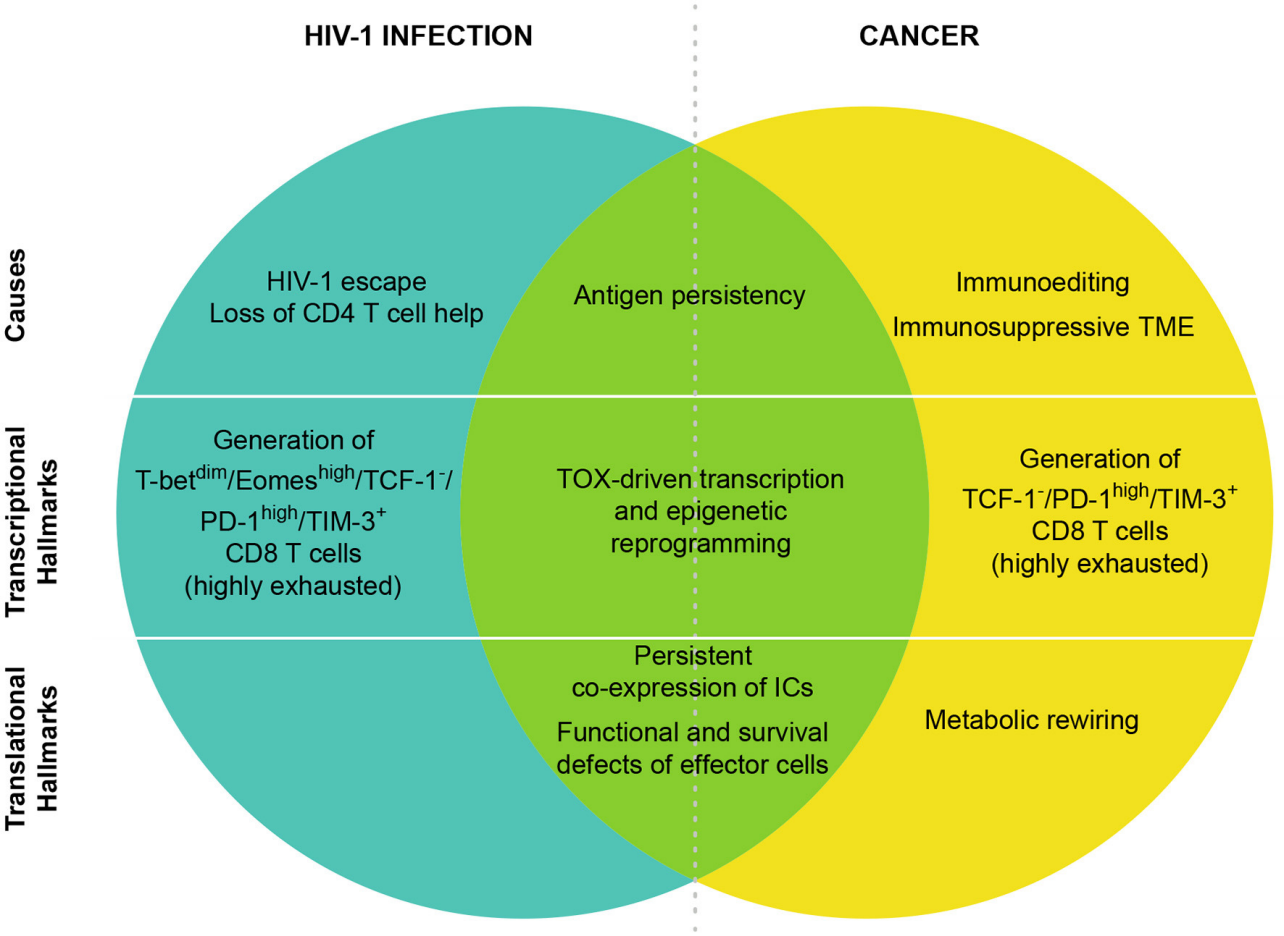
CD8 T cell exhaustion in HIV-1 and cancer. T cell exhaustion in HIV-1 infection and cancer presents common origins and hallmarks, but also different features. The shared cause of T cell exhaustion is antigen persistency due to immune escape mechanisms. Moreover, in HIV-1 infection, the high viral mutational rate contributes to the immune escape while the preferential tropism of the virus for HIV-1 specific CD4 T cells leads to CD4 T cell loss that is also a main contributor of CD8 T cell exhaustion. In cancer, immunoediting and TME immunosuppression are peculiar determinants of tumor-specific CD8 T cell exhaustion. In both cancer and HIV-1 infection TOX has been identified as a master regulator of the transcriptional and epigenetic reprogramming of exhausted T cells. In HIV-1, T-bet^dim^/Eomes^high^ subset defines highly exhausted CD8 T cells, however in CD8 T cells from cancer patients T-bet and Eomes are expressed in T cells with different levels of exhaustion. At the protein level, the co-expression of many ICs has been identified as hallmark of T cell exhaustion in both cancer and HIV-1 infection. Exhausted cells are also characterized by functional and survival defects including reduced effector functions, expansion capacity and increased susceptibility to apoptosis. Metabolic rewiring is also a key player of T cell exhaustion, however in HIV-1-infected patients little/no information is available so far.

Induction of T cell exhaustion is a common trait between HIV-1 infection and cancer (17), however key differences distinguish antiviral from anti-tumor immunity due to the pathogenesis of the two diseases (Figure 1 and Table 1). The immunogenicity of the tumor is shaped by the immune system through a process called “immunoediting,” as the pivotal work of Bob Schreiber first showed 15 years ago (116). In a first phase, the adaptive and the innate immune systems synergize to recognize and eliminate malignant cells using conventional mechanisms (elimination phase). These include: the specific recognition of tumor-associated antigens and the expression of effector molecules by T lymphocytes (type I and II- interferon, perforin, Fas/FasL, tumor necrosis factor (TNF)-related apoptosis-inducing ligand—TRAIL), analogously to a viral infection, paralleled by the expression of recognition molecules such as NKG2D or ligands on tumor cells (induced by DNA damage and stress pathways) (117). Early infiltration of tumors by immune cells such as pro-inflammatory macrophages, both CD4 and CD8 T lymphocytes, NK, and DCs is crucial for tumor control (118–121). In the second phase, dormant tumor cells survive in equilibrium with the immune system where immunosuppressive and anti-tumor functions are balanced (equilibrium phase). In this phase, the tumor microenvironment (TME) is composed of several cell types that produce variable amounts of immune-suppressing and immune-stimulating molecules. In addition, tumors show a low proliferation rate and progressively undergo editing, resulting in tumor cell variants able to escape immune control (escape phase) (122). Clinically detectable tumors belong to this last and most studied phase with cancer cells proliferating with no or limited constraints. Tumor cells directly induce T cell exhaustion through the acquisition of somatic mutations, which confer increased immune resistance and survival, altogether contributing to prolonged antigen exposure. Ultimately, the exhaustion state is the outcome of a transcriptional and metabolic reprogramming induced by immunosuppressive cytokines (i.e., TGF-β, IL-10) and metabolites (i.e., lactate, kynurenine, adenosine, PGE2) produced by cancer cells (121, 123) and tumor infiltrating immunosuppressive cell subsets, including regulatory T cells, myeloid-derived suppressor cells, tumor-associated macrophages, cancer-associated fibroblasts, adipocytes, and endothelial cells (124). Moreover, anti-tumor T cells compete with cancer and immunosuppressive cells for nutrient availability and immunostimulatory factors. Current data suggest that nutrient deprivation is inducing, *per se*, T cell dysfunction (125–127).

**Table 1:** Hallmarks of exhaustion.

**IC expression**	
**HIV-1/chronic infection**	**Cancer**
PD-1 (23–25)	PD-1 (51–56)
CTLA-4 (57)	CTLA-4 (53)
TIM-3 (34, 58–60)	TIM-3 (52, 53)
LAG-3 (61)	LAG-3 (53)
TIGIT (62, 63)	TIGIT (64–66)
CD160 (33, 67, 68)	CD160 (69)
2B4 (CD244) (67, 68)	2B4 (CD244) (70)
BTLA (60)	BTLA (71)
CD6 (72)	KLRG1 (73)
	VISTA (74–76)
	CD39 (77, 78)
	CXCL13 (53, 79)
	LAYN (80)
	Sia-SAMP:Siglec-9 (81)
**Transcription factors expressed by exhausted CD8 T cells**	
**HIV-1/chronic infection**	**Cancer**
Master regulators: TOX, TCF-1 (82–85)	
EOMES 187 (85)	STAT3 (86, 87)
BLIMP-1 (88–91)	BLIMP1 (55, 92)
TOX (93)	TOX (64, 93)
NOTCH (94)	NR4A2 (95)
NFATc1 (96)	NFAT (95)
BATF (97–100)	BATF (55)
IRF4 (100)	IRF4 (85)
VHL (101)	VHL (101)
FOXO1 (102)	FOXO1(102)
PBX3 (19)	FOXP1 (103)
c-Myb (85)	cMAF (104)
	GATA-3 (105)
	Zinc-dependent TFs (105)
**Epigenetic of exhaustion**	
**HIV-1/chronic infection**	**Cancer**
PD-1 locus demethylation was observed in models of chronic infections (106) and in HIV-1 infected patients (107)	Tumor-reactive makers CD39 and CD103 are demethylated in tumor-reactive CD8 T cells (whole-genome methylation profiling) (108)
Increased accessibility to *Pdcd, Havcr2*, and *Batf loci* and to *loci* encoding genes involved in negative regulation of T cell effector functions (109)	Recurrence after anti-PD-1 therapy was associated with the hypermethylation of the PD-L1 promoter (110)
Recent studies show the stability of the PD-1 locus demethylation even after PD-1 blockade (111)	Two chromatin states have been identified in exhausted T cells: (i) plastic and reversible, (ii) fixed dysfunctional state resistant to reprogramming (112)
Identification of exhaustion-specific enhancer that contains essential RAR, T-bet, and Sox3 motifs (109)	HDAC6-selective inhibitors directed peripheral and infiltrating T cells toward a Th1/effector phenotype (113)
Exhausted T cells acquire heritable *de novo* methylation programs able to restrict T cell expansion and clonal diversity during PD-1 blockade treatment. A DNA-demethylating agent (Decitabine) improved T cell responses and tumor control during PD-1/PD-L1 blockade (114)	
9–12 exhaustion clusters have been identified from epigenomic-guided mass cytometry profiling data (115)	

CD8 T cell responses are quickly impaired during both early viral infection and tumor establishment.

Recently, terminally exhausted CD8 T cells have been characterized and distinguished from their progenitors depending upon the expression of PD-1, TIM-3, CD44, Eomes, T-bet, TCF-1, Slamf6, and CXCR5 (51, 67, 128–134). Exhausted T cell progenitors were characterized in LCMV models as pool of cells expressing TCF-1^+^/PD-1^int^/CXCR5^+^/Slamf6^+^, responding to PD-1 blockade and differentiating into terminally exhausted CD8 T cells (TCF-1^−^/PD-1^high^/TIM-3^+^) (128–131). Their presence was also described among circulating tumor-reactive CD8 T cells in melanoma patients and within TILs in primary melanomas (135) and non-small-cell lung cancer (NSCLC) (132). Interestingly, recent studies have better characterized a subset of CD8^+^/CXCR5^+^ T cells with proliferative capacity and able to infiltrate B cell follicles and inflamed tissues in the presence of chronic antigen exposure and inflammation (129, 131, 136–143). This subset shows heterogeneous phenotype and gene expression profile depending on the pathogenic context, still it is distinct from the CXCR5^−^ counterpart pool and maintain cytotoxic properties (144). In addition of being part of the TCF-1^+^/PD-1^int^ progenitor pool (129, 145), these cells have been described as having variable levels of exhaustion and being similar to Tfh cells (20, 108, 129, 131, 141, 146–149). This is reflected in their capacity to help in the control of viral infection and of tumor growth, in the promotion of inflammation and in the induction of B cell responses (108, 136, 137, 144). The formation and maintenance of the TCF-1^+^/PD-1^int^ progenitor pool is orchestrated by the thymocyte selection-associated high mobility group box protein TOX. While TOX is a key player in the establishment of the exhausted state, its role is largely dispensable for the generation of effector and memory T cells. Antigen persistency is likely to be the cause of *Tox* induction since its expression is dependent on calcineurin and NFAT2. TOX is therefore the translator of persistent stimulation into a distinct T cell transcriptional and epigenetic developmental program leading to T cell exhaustion. TOX is also important for the subsequent differentiation into terminally exhausted cells that is counteracted and regulated by the phosphatase PTPN2 (82–84, 150–152). PTPN2 abrogation increases the number of terminally differentiated cytotoxic CD8 T cells promoting effective immune response, tumor/viral clearance and improved response to inhibitory molecules blockade (84). TOX induces genes that are important for the exhaustion precursor formation, including transcription factors (TFs) (e.g., *Tcf7, Nr4a2*, and *Tox* itself) and co-inhibitory receptors (e.g., *Pdcd1, Lag3, CD244*, and *Havcr2*). In conclusion, persistent activation and induction of TOX are common drivers of T cell exhaustion in both viral infection and tumor pathogenesis. However, specific players such as CD4 T cell loss and TME heterogeneity in infection and cancer, respectively, contribute to define distinct and overlapping traits of exhausted T cells in the two conditions.

## Hallmarks of T Cell Exhaustion in HIV-1 Infection

Many studies have indicated HIV-1-induced T cell exhaustion as main hallmark of the disease. Of note, HIV-1-specific CD8 T cells selectively show features of exhaustion as compared to bulk CD8 T cell populations and unrelated virus-specific T cells circulating in the same subject, as described in human and animal studies (153–155).

### Expression of Multiple ICs

A complex network of stimulatory and inhibitory surface molecules orchestrates the functionality of CD8 T cells (156, 157). A cardinal feature of exhausted T cells in HIV-1 infection is the sustained expression of multiple inhibitory immune checkpoints (ICs) (Table 1).

The first and, to date, the most important IC involved in CD8 T cell exhaustion in chronic infections (15, 23–25, 52, 158) is PD-1. During chronic stimulation, PD-1 expression on virus-specific CD8 T cells is high and sustained (23–25, 68) because of mechanisms involving both TFs [i.e., T-bet (159), Blimp-1 (88, 160)] and soluble factors [i.e., IFN-α (161) and RANTES (156)]. In turn, PD-1 signaling affects the function, proliferation, survival and chemotaxis of CD8 T cells (23–25, 156, 162). *In vivo*, PD-1^high^ SIV-specific CD8 T cells are characterized by a higher turnover (163).

The interaction of PD-1 with its two ligands PD-L1 and PD-L2 on hematopoietic and non-hematopoietic cells triggers the phosphorylation of two cytoplasmic domains and the subsequent recruitment of cytosolic tyrosine phosphatases Shp2 and Shp1, the TCR-phosphorylating kinase Lck, and the inhibitory tyrosine kinase Csk (164, 165). These effectors mainly act by antagonizing the CD28 costimulatory signaling (166–168) and the TCR signaling *via* dephosphorylation of SLP76 and ZAP70 (164, 166). Moreover, signaling molecules including ERK, Vav, PLCγ, PI3K, and Ras have been described as downstream targets of PD-1 signaling in T cells, leading to an impairment in metabolism, survival and cell growth (10, 165, 168, 169). PD-1 is also expressed by CXCR5^+^ CD8 T cells (20, 170), a population particularly interesting for therapeutic purposes.

In addition, landmark studies in LCMV (67) and then SIV/HIV-1 infection (33, 34, 62, 171, 172) highlighted the relevance of multiple ICs co-expression (i.e., CD160, 2B4, TIM-3, T cell immunoreceptor with Ig and ITIM domains-TIGIT, CTLA-4 and LAG-3) to define deeply exhausted virus-specific CD8 T cells. The co-expression of multiple ICs may be due to their transcriptional co-regulation and non-redundant roles in the physiological control of CD8 T cell responses (130, 173–176). Increased disease progression, viral replication and lower CD4 T cell counts were directly associated with PD-1 (23), CTLA-4 (171), TIM-3 (58, 59), LAG-3 (61), and TIGIT (62, 63) expression. In addition, the superior proliferative capacity and the maintenance of cytotoxic functions by CXCR5^+^ CD8 T cells concur with a lower surface expression of ICs and a higher expression of co-stimulatory receptors (CD28 and ICOS) as opposed to the CXCR5^−^ counterpart (129, 131, 148). Of importance, SIV and HIV-1 specific CD8 T cell proliferation *in vitro* improves when distinct ICs (*i.e*. CD160, 2B4, TIGIT, BTLA, TIM-3) are blocked (24, 33, 60, 62) and administration of anti-PD-1 in SIV infected macaques (177–181) and HIV-1-infected patients (182) increases T cell immune responses, however clinical efficacy remains controversial (181, 183–190).

More recently, in SIV-infected macaques, the expression of CD6 by PD-1^+^ CD8 T cells was associated with a reduced proliferation, cytokine secretion and cytotoxic capacity when compared to their CD6^−^ counterpart. The frequency of CD6^+^PD-1^+^ CD8 T cells positively correlated with SIV viral load and combined targeting of CD6 and PD-1 effectively restored the CD8 T cell proliferation capacity *in vitro*, suggesting that CD6 may be a new immunotherapeutic target (72).

Recently, the combination of transcriptomic and proteomic data allowed the identification of multiple cell clusters that were evolving with HIV-1 disease progression or initiation of ART (64). These data may lead to the understanding of new specific features of disease evolution and drive novel therapeutic approaches.

### Alteration in TFs Expression and Epigenetic Regulation

Genomic approaches were recently applied to investigate the transcriptional profile of virus-specific exhausted CD8 T cells, revealing their unique molecular signature as compared to non-exhausted cells (Table 1) (19, 109, 111, 112, 115). Transcriptional analyses showed that exhaustion results from centrally connected pathways (19, 115, 191), having TOX as a master regulator. Indeed, TOX expression correlates with the presence of an exhausted phenotype during chronic infections in mice (LCMV) and humans (HCV) (82). In addition to TOX, several TFs coordinate gene expression networks, including PBX3, EOMES, BLIMP1 (*Prdm1*) (88–91), NOTCH (94), NFATc1 (96), basic leucine zipper transcription factor, ATF-like (BATF) (97–99), IRF-4, von Hippel–Lindau disease tumor suppressor (VHL), FOXO1, and FOXP1 (99–102, 130, 159, 192–198). At the molecular level, TCR stimulation leads to the induction of *Tox* expression (83) and induces the recruitment of TFs, like Notch (94), NFATc-1 (96), IRF-4 and BATF (100), at the promoter of different inhibitory receptors, ultimately driving their upregulation. Among the genes induced by TOX, *Tcf7* (encoding TCF-1) promotes the generation of exhaustion precursors through the induction of *Eomes* and *c-Myb* in early chronic infection, whereas PD-1 is needed to stabilize this pool (85, 199). IRF4 was also shown to favor CD8 T cell exhaustion while limiting memory T cell differentiation (100). Importantly, PD-1^high^/Eomes^high^ and PD-1^low^/T-bet^high^ T cells are both necessary to contain chronic LCMV infection (130). However, CD8 T cells presenting a T-bet^dim^/Eomes^high^ profile represent a highly exhausted state with elevated levels of multiple inhibitory receptors (i.e., PD-1, CD160, and 2B4) (200, 201). In turn, PD-1 signaling reduces the expression of Bcl-xl (168), favoring the apoptosis of activated T cells (162, 202), and induces BATF leading to a decreased cytokine production, cytotoxic potential and proliferation rate of virus-specific CD8 T cells (97, 99). BATF induces the expression of T-bet and BLIMP-1 and correlates with PD-1 expression in murine models of chronic viral infection. BLIMP-1 is upregulated in patients with progressive, as opposed to non-progressive, HIV-1 infection (194, 203, 204) and is also associated with reduced T cell proliferation and effector-cytokine secretion capacity; however, these functions are restored by knocking down BATF or BLIMP-1 (88, 99). BLIMP-1 can also be induced in T cells upon priming with HIV-1 pulsed DCs together with other inhibitory molecules, including PD-1, TIM-3, LAG-3, and CTLA-4 (205).

**Figure 2:**
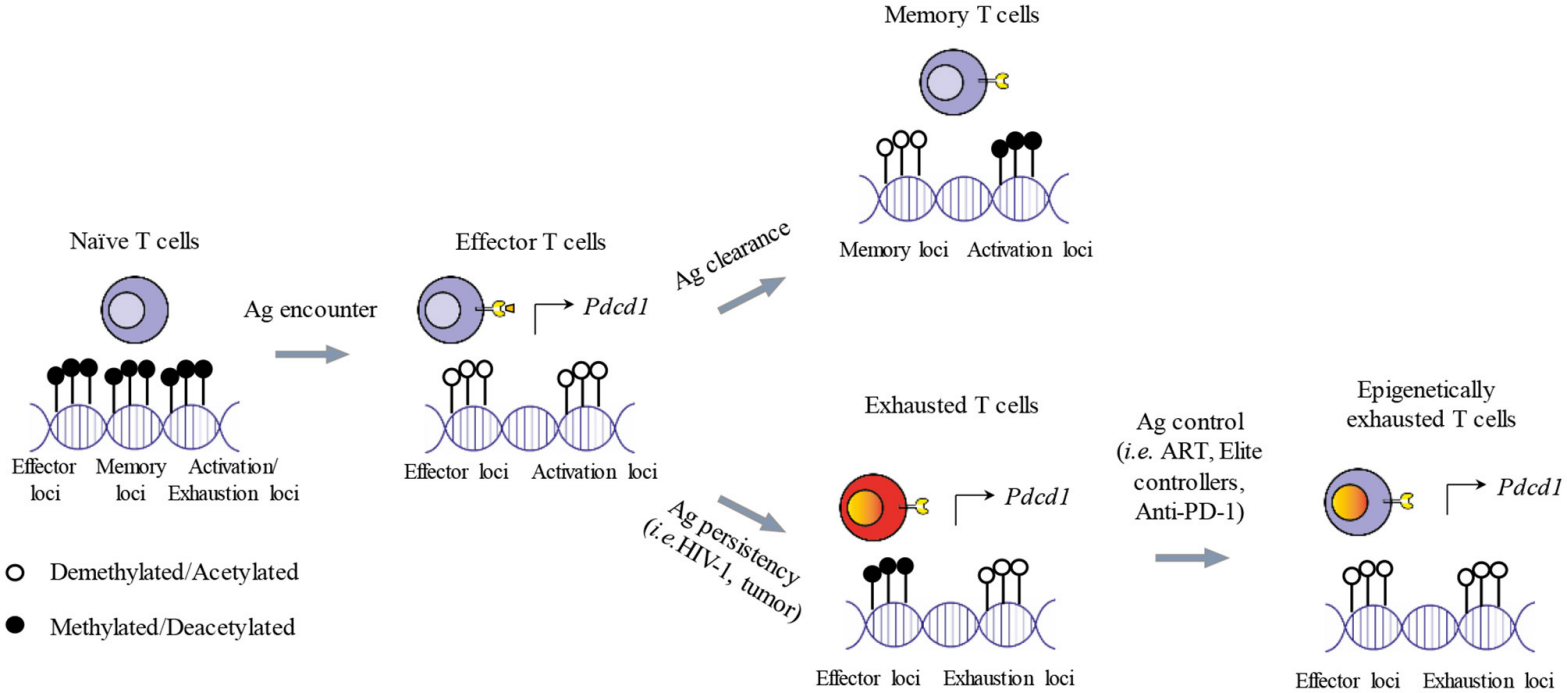
Epigenetic imprinting of T cell exhaustion. Shortly after antigen exposure, naïve T cells generate effector T cells that are armed to eliminate foreigner antigens. Activated T cells demethlyate *loci* dedicated to the expression of effector functions and activation genes. Activated genes include also ICs (i.e., PD-1) needed for starting the contraction phase once the antigen is cleared. After the contraction phase, memory T cells survive and present a specific transcriptional profile, while PD-1 expression is reduced. If the antigen persists, memory T cells cannot be generated and effector functions are progressively lost. In addition, some genes of the demethylated *loci* remain transcriptionally active sustaining the expression of ICs and leading to T cell functional exhaustion. In the context of a therapeutic intervention or physiological immune control of chronic antigen exposure (i.e., ART, Anti-PD-1, Elite controllers), exhausted T cells can restore, at least partially, their effector functions and reduce the expression of ICs such as PD-1. However, implicated *loci* remain demethylated, potentially causing a rapid restoration of the exhausted state after treatment interruption and the failure of the immune system to completely eradicate the antigens.

The characterization of the epigenetic landscape of exhausted T cells gives novel and key insights to decipher the function of TFs. Comprehensive whole-genome analysis of chromatin accessibility (ATAC-seq) (206), has shown that exhausted CD8 T cells have a distinct epigenetic signature (95, 109, 111, 112, 207, 208). For instance, exhausted CD8 T cells have several chromatin regions with reduced accessibility (e.g., the *Ifng, Ccr7, Il7r, Nt5e, Tcf7*, and *Lef1 loci*), while presenting open chromatin regions in loci that govern the expression of IC molecules (e.g., *Pdcd1, Tigit, Ctla4)*, of ectoenzymes implicated in metabolic regulation (e.g., *Cd38, Entpd1*), of chemokines and cytokines (e.g., *Xcl1*) and of TFs (e.g., *Eomes, Ikzf2, Tox*) (64, 109, 111). The deletion of chromatin accessible regions including TF binding motif for RAR-retinoic acid receptor, T-bet, and Sox3 cause a dramatic reduction in PD-1 expression, demonstrating their important role in shaping exhausted T cell transcriptional profiling (64, 109, 208). Moreover, during chronic LCMV infection, the *Pdcd1* locus become completely demethylated (106), while the histone H3 is less diacetylated in CD8 T cells, indicating a loss in epigenetically active genes (209). In parallel, the transcriptional regulatory region of the PD-1 promoter is unmethylated in PD-1^hi^ HIV-1-specific CD8 T cells but not in donor-matched naive cells (PD-1^−^) (107). Thus, in chronic LCMV (106) and HIV-1 infection (107), PD-1 expression in virus-specific CD8 T cells is controlled by the chromatin accessibility of the gene itself (epigenetic control) and by TF governing its expression (Figure 2).

### Loss of Functions

Exhaustion in chronic viral infections has been described in both mice and humans as the progressive decrease in the capacity of virus-specific CD8 T cells to secrete cytokines, proliferate and exert cytotoxicity (23, 68, 210–213) as a consequence of persisting virus and antigen load (214). Loss of function characterizing exhaustion is hierarchical: IL-2 production is one of the first function to be extinguished, followed by TNF-α production, whereas the ability to produce interferon-γ (IFN-γ) is more resistant to inactivation (155, 213, 215–218).

Consistently with the hierarchical loss of effector functions by exhausted T cells, Riley et al. (219) demonstrated that such effector functions depend on the strength of PD-1 signaling, thus on PD-1 expression levels.

## Hallmarks of T Cell Exhaustion in Cancer

The identification of exhausted T cells in the cancer setting was inspired by previous knowledge gained in chronic viral infections. In human melanoma metastasis, T cells sharing many features of the exhaustion signature identified in LCMV infection were found (53). However, as discussed above, the establishment of exhaustion occurs differently in viral infection and cancer, the latter involving a complex network of players and mediators. The repertoire of tumor-specific T lymphocytes is generally devoid of highly avid autoreactive cells due to central and peripheral tolerance mechanisms, and priming may be inefficient due to the lack of co-stimulation, an inflammatory milieu and/or the presence of immunoregulatory cellular subsets (220). Therefore, a more heterogeneous pool of cells, fully activated or not, may undergo the dysfunctional program. Consistently with their virus-specific counterparts, these cells are characterized by increased expression of ICs (19, 64, 221), impaired homeostatic response to cytokines (222) and altered epigenetic and transcriptional programs (10, 191, 223). In contrast to HIV-1 infection where little/no information is available to date, the rewiring of the T cell metabolism in cancer immunopathogenesis is a well-characterized hallmark of exhaustion (224, 225). Of note, PD-L1 engaged by PD-1 acts as an anti-apoptotic molecule and increases chemoresistance on cancer cells through phosphorylation and activation of the PI3K/AKT pathway, as opposed to inactivation in T cells (226–228). Notwithstanding the recent burst of investigations on T cell exhaustion in cancer, studies in human remain challenging and animal models should be tuned to better reflect the slow course of natural cancer progression and its antigenic contexts (high/low mutational load).

### Expression of Multiple ICs

In line with what is described for HIV-1 infection, a high and sustained expression of ICs is consensually considered as the main hallmark of T cell exhaustion in the cancer setting (Table 1). Tumor-specific CD8 TILs express high levels of PD-1 associated to impaired function (54). PD-1 is expressed upon TCR engagement and NFAT nuclear translocation (96) and may drive exhaustion of T cells undergoing persistent antigen exposure (18, 229). Exhausted T cells can co-express PD-1 together with different ICs, including, LAG-3, CTLA-4, BTLA, TIGIT, 2B4 (CD244), VISTA, KLRG1 (53, 73, 230) and TIM-3 (52, 131, 199). Inhibitory receptors signal through non-overlapping pathways and use different mechanisms to regulate T cell function ultimately inducing exhaustion: they sequester target receptors and ligands involved in activation pathways (ectodomain competition), they dampen the signals from activating receptors and they mediate transcription of inhibitory genes (10). Importantly, the hierarchical co-expression of multiple inhibitory receptors has been associated with a more severe grade of cellular dysfunction (231). Additional, recently identified markers of CD8 T cell exhaustion in cancer include: CD39 (77, 78), LAYN, whose expression is mutually exclusive with LAG-3 in hepatocellular carcinoma patients (80), and CXCL13 (53, 79). Moreover, Stanczak et al. (81) described the Sia-SAMP:Siglec-9 as an inhibitory pathway in NSCLC, where high frequencies of Siglec9^+^CD8^+^ TILs inversely correlate with survival (81). Finally, CD160^+^ CD8 T cells have been shown to express higher PD-1 levels than the CD160^−^ counterpart, to have less proliferative and cytotoxic potential and to be enriched among CD8 TILs in pancreatic cancer patients (69). Recently, a CXCR5^+^ CD8 T cell population has been observed to expand in diffuse large B cell lymphoma (232), follicular lymphoma (144) and HBV-related hepatocellular carcinoma (137, 139, 141, 149). Circulating, tumor infiltrating, and lymphoid CXCR5^+^ CD8 T cells were shown to co-express PD-1 and, in contrast with chronic viral infection (129, 131, 134, 148), TIM-3 (134, 140), however they were functionally less exhausted than the CXCR5^−^ CD8 T cell population and expressed genes related to stem-like plasticity and cytotoxicity (140, 141, 149). The frequency of this subset was correlated with a better prognosis in follicular lymphoma (144), pancreatic (139), colorectal (137, 141), and lung (140) cancer, suggesting its anti-tumor activity. However, combined blockade of TIM-3, PD-1 or IL-10R pathways could increase the cytotoxic activity of CXCR5^+^ CD8 T cells indicating their limited lytic potential (139, 149).

### Alteration in TFs Expression and Epigenetic Regulation

Tumor cells, together with immune and non-immune populations of the TME, contribute to a well-defined gene expression profile of dysfunctional anti-tumor T cells (Table 1), partially overlapping with that of exhausted T cells in chronic infections, by releasing molecules and establishing inhibitory contacts. In addition, recent studies in murine and human cancer suggest that TILs display a broad spectrum of dysfunctional states shaped by the multifaceted suppressive signals that occur within the TME (64, 130, 135). Several signaling pathways through the TCR, suppressive cytokines (TGF-ß, IL-6), inhibitory receptors, metabolites (adenosine, prostaglandins, lactate), enzymes (e.g., nitric oxide synthase, reactive oxygen species, indoleamine-2,3 dioxygenase), low pH, hypoxia and nutrient deprivation, lead to the final transactivation of TFs controlling the expression of different gene sets (101, 104, 173, 233, 234). As described for chronic infections, a complex pattern of TFs drives the initial triggering of differentiation toward the exhausted phenotype, including TOX, NFAT, Blimp-1, BATF, FoxO1, VHL, IRF4 (93, 234), Bcl-6, cMAF, and STAT3 (86, 87, 104, 235, 236). These factors exert distinct roles in T cells at different stages of differentiation and they do not exclusively govern gene expression in exhausted T cells. The epigenomes of different T cell subsets contribute to the context-specific functions of shared TFs. For instance, STAT3 dependent transcriptional regulation limits both TILs recruitment and cytotoxic function by downregulating IFN-γ, CXCR3, and CXCL10 expression and inducing ROR-γt (87, 236). Of note, EOMES and T-bet are expressed during the whole course of tumor progression and, in contrast to chronic viral infections, they do not help in distinguishing an exhausted-progenitor subset from terminally differentiated exhausted T cells (Figure 1) (10, 55). More recently, new technological advances (*i.e*., mass cytometry and single cell sequencing) are allowing a deeper examination of the molecular properties of dysfunctional T cells at the single cell level. These studies represent milestones for the comprehension of T cell biology in the context of complex TME, dominated by a high heterogeneity of cellular subsets. Recently, Bengsch et al. (64, 115) identified 9 distinct T cell clusters among exhausted CD8 T cells in HIV-1 infection and human lung cancer by using transcriptomic- and epigenetic-guided mass cytometry. This study also assigned an exhaustion score to each of the subsets based on functional features (64, 115), those providing relevant insight for the design of IC blockade therapies.

### Loss of Functions

As in chronic viral infections, exhausted T cells found in different tumor types have reduced effector functions as shown in terms of cytokine production and cytotoxicity (53, 237). Nevertheless, the hierarchy by which T cells progressively lose their functions is less clear (53, 54, 231, 237–239). TILs are not functionally inert and, to some extent, contribute to tumor control (231, 240). The efficacy of IC inhibitors and IL-2-driven *ex vivo* expansion of functional TILs is an indirect proof of this impaired yet present anti-tumor activity. Furthermore, TILs can be highly heterogeneous among distinct cancer types as evidenced by their different capacity to respond to IC blockade. For instance, in small-cell lung cancer patients, subsets of PD-1^high^ TILs are enriched in tumor-specific T cells and their presence is a predictor of clinical response to anti-PD-1 therapy (132, 241–243). On the contrary, T cells infiltrating breast tumor retain robust cytokine production and degranulation capacity (244) notwithstanding the expression of PD-1. In breast cancer patients, PD-1 expression is therefore less predictive of TILs dysfunction and this may explain the modest clinical responses to anti-PD-1 or anti-PDL therapies.

The proliferative potential of exhausted T cells is considered limited due to unresponsiveness to homeostatic cytokines such as IL-7, IL-15 and IL-21 (211, 245, 246). However, the previously mentioned TCF-1^+^/ PD-1^int^ progenitor pool of exhausted T cells has a residual proliferative potential that allows the replenishment of the pool of exhausted antigen-specific CD8 T cells by expanding and differentiating into the numerically larger population of TCF-1^−^/PD-1^hi^/TIM-3^+^ terminal progeny, characterized by a higher co-expression of other ICs and limited proliferative capacity (135, 199).

In a work by Li H. and co-workers, the intra-tumoral immune infiltrates of 25 melanoma patients differing for staging and treatments were analyzed by scRNA-seq for a deep characterization of dysfunctional T cells both in terms of transcriptional states and TCR clonality (238). Exhausted T cells expressing previously reported ICs (i.e., PD-1 and LAG-3) were observed in many patients. Importantly, intra-tumoral CD8 T cells could cluster in two distinct subpools. T cells belonging to the first pool spanned a wide range of transcriptional states, from transitioning to highly dysfunctional, expressed a gradient of inhibitory molecules and were specifically observed in tumor tissue. Some of the expressed regulatory molecules (CSF1, ZBED2) were also shared with regulatory T cells. A second subpool included T cells with cytotoxic potential, but limited proliferative capacity. This second pool of T cells could represent bystander T cells, likely from the circulation. Tumor-specific T cells were enriched in the exhausted pool, as previously observed for NSCLC (132). Strikingly, T cells with an initial buildup of the dysfunctional program maintained a clear proliferative signal with a doubling time of few days and rapid turnover. This dynamic and active T cell state fits previously suggested models of establishment of exhaustion at the tumor site (238). Common mechanisms of the emergence of exhaustion are present among tumor types, but differences in the relative abundance of the subsets can be due to different TME, i.e., availability of antigens and exposure to inhibitory factors as well-shown for TILs in breast cancer (244). This is then reflected in the different capacity to respond to IC blokade that is not only heterogeneous among tumor types but also among individuals (244), as reviewed elsewhere (247).

In conclusion, in both HIV-1 infection and established tumors, T cell exhaustion is likely driven by TOX and the subsequent coordinated expression of several TFs. Exhausted T cells are characterized by loss of effector functions, high expression of multiple ICs, reduced homeostatic expansion, altered TFs expression, and remodeled chromatin. However, while in HIV-1 infection T-bet and EOMES allow the distinction between progenitors and fully exhausted T cells, in cancer patients TCF-1 and STAT3 may instead be the key TFs. The avidity and the hierarchy of the loss of function of exhausted T cells in cancer patients is less well-described than in chronic infections. Exhausted cells present in the TME may be highly heterogeneous and not include only the antigen-specific ones; new insights will explain how these aspects could affect the response to IC blockade.

## Exhausted vs. Activated/Memory CD8 T Cells

Given the high heterogeneity and dynamicity of the memory CD8 T cell compartment (64, 238, 248), novel immunotherapies, aiming at rescuing the functionality of exhausted T cells, would require the ability to selectively distinguish exhausted from memory and activated effector T cells.

### Expression of Surface Molecules

The solely qualitative evaluation of ICs expression by CD8 T cells, *per se*, does not discriminate between exhausted and activated T cells. As previously mentioned, inhibitory receptors that are transiently expressed on activated effector T cells show a higher and sustained upregulation on exhausted T cells, triggered by a persistent antigen stimulation. For instance, PD-1 is rapidly upregulated upon T cell activation (249) and persists at moderate levels in healthy subjects with a preferential expression on effector memory T cells (162, 250–253). During chronic infections, PD-1 expression on viral-specific T cells increases (23, 38, 128, 254, 255) and does not always reverse upon antigen removal (175, 256). HIV-1-infected patients responding to ART show reduced expression levels of PD-1 on virus-specific CD8 T cells after antigen clearance (257), still these levels are maintained above the physiological threshold observed in healthy individuals. This may be due to a broad systemic immune activation, to the effects of common gamma-delta chain cytokines sustaining PD-1 expression on bulk CD8 T cells (258, 259) or to the irreversible transcriptional and epigenetic alteration affecting highly exhausted T cells (106, 260) (Figure 2).

As previously mentioned, the degree of exhaustion is directly associated with the pattern of co-expression of different co-inhibitory receptors (67). First, this is mechanistically relevant, as simultaneous blocking of multiple ICs results in a synergistic reversal of T cell exhaustion in both cancer and chronic infections (171, 239, 261–263). Second, the identification of co-expression subsets may lead to a better discrimination between exhausted and activated T cells, reducing the risk for off-targets effects.

Many studies have shown that chronic antigen stimulation of T cells drives an IC expression pattern. For instance, TIM-3 and PD-1 cooperate for the induction of CD8 T cell exhaustion in cancer (52, 264–266) and chronic viral infections (34). In LCMV infection, PD-1 and TIM-3 identify a population of T cells strongly enriched in gene signatures of terminal exhaustion and harboring reduced proliferative capacity, longevity and cytokine production (64). In HIV-1 infected patients, ART significantly suppresses TIM-3 expression on HIV-1 specific CD8 T cells (267) indicating that, like PD-1, it is dependent on chronic TCR stimulation. Moreover, the expression profile of CD56 and TIM-3 can discriminate between individuals that naturally control HIV-1 replication (elite patients) and ART-treated patients (268). After virus-clearance and CD4 T cell recovery, patients receiving ART show a quantitative loss of CD56^+^ CD8 T cells coupled to an exhausted phenotype, as shown by TIM-3 upregulation. Elite patients maintain a pool of cytolytic CD56^+^ CD8 T cells comparable to healthy individuals. Similarly, CD160 expression also allows the distinction between exhausted and activated (PD-1^+^) HIV-1 specific CD8 T cells (33). Indeed, only cells co-expressing CD160 and PD-1 (PD-1^high^CD160^high^) are functionally impaired in HIV-1 infected patients (33).

In cancer, the activation of ICs other than PD-1/PD-L1 and CTLA-4 can be induced by adaptive resistance to IC therapies. The treatment of such tumors could benefit from the combination of anti-PD-1 with different immune checkpoint molecules (e.g., LAG-3, TIM-3, TIGIT), activation markers and cytokines/chemokines (269). On the other hand, T cell dysfunction is characterized by decreased levels of co-stimulatory molecules, of their ligands and of adaptor molecules impairing the co-stimulatory signaling. Among these, CD44, LY6C, killer cell lectin-like receptor subfamily G member 1 (KLRG1), CD122 (IL-2Rβ), and CD127 (IL-7R), tumor necrosis factor receptor (TNFR)- associated factor 1 (TRAF1), CD28, and 41BBL have been described (67, 175, 246, 258, 259). In particular, exhausted T cells display the same profile of effector T cells with reduced telomere length and low levels of CD62L, CD127, and CD122 expression (1, 40, 153, 215, 258, 270–273). Their incapacity to respond to IL-7 and IL-15 (88, 128, 159, 245) lead to the lack of homeostatic expansion in the absence of antigens (1, 88, 128, 130, 159) and, ultimately, to death (14, 154, 215, 256, 274–276). T cell dysfunction is also characterized by the downregulation of the signaling adaptor TNFR-associated factor 1 (TRAF1) both in HIV-1 infected patients with progressive disease and in LCMV chronically-infected mice (259). In HIV-1 infected patients, TRAF1 expression negatively correlates with PD-1 expression and viral load and knockdown of TRAF1 in CD8 T cells from viral controllers results in decreased HIV-1 suppression *ex vivo*. TGF-β is responsible for the post-translational loss of TRAF1, while IL-7 signaling is able to restore TRAF1 levels. Transfer of TRAF1^+^ memory T cells or a combination treatment with IL-7 and agonist anti-4-1BB antibody in chronic LCMV infection improve T cell expansion and viral control in a TRAF1-dependent manner (259). Patient samples of renal cell carcinoma also show reduced expression of TRAF1 compared with normal kidney. This confers resistance to apoptosis and higher proliferative capacity to renal cancer cells (277). These findings identify TRAF1 as a potential biomarker of T cell dysfunction and therapeutic target. Moreover, combining PD-1 blockade with an agonistic antibody to 4-1BB dramatically improved T cell function and LCMV control *in vivo* (278). Still, the role of positive co-stimulatory molecules in rescuing exhausted T cells remains poorly described.

### Transcriptional and Epigenetic Regulation

Another key difference between exhausted and activated T cells resides in the TFs (18, 19, 191, 279). Both the quality of the expressed TFs and the genes they can target, distinguish exhausted T cells from activated and memory CD8 T cells (191, 280, 281).

Transcriptional profiling analysis demonstrated that CD8 T cell memory and exhaustion reflect distinct states defined by coordinated sets of modules. Specific genes and pathways differentially implicated in exhaustion *vs*. memory include genes involved in epigenetics, DNA damage, and WNT signaling, such as *Rtp4, Foxp1, Ikzf2, Zeb2, Lass6, Tox*, and *Eomes* (191). The study by Bengsch et al. (64, 115) associates effector and exhausted T cells to a higher expression of CD39, LAG-3, TCF-1, Helios, CTLA-4 and PD-1, Eomes, TOX, 2B4, TIGIT, respectively.

During acute infection, T-bet and EOMES play pivotal roles in the generation of terminally-differentiated (2, 282) and central-memory (283–285) CD8 T cells respectively, while CD8 effector T cells co-express T-bet and EOMES (286). In contrast, during chronic infection, exhausted T cell subsets express either T-bet or EOMES in a somehow mutually exclusive pattern and they identify pools of non-terminal progenitor and terminally-differentiated exhausted CD8 T cells, respectively (Figure 1) (130). Of note, anti-PDL1 therapy only improves the function of the T-bet^hi^ subset, while having little impact on EOMES^hi^ cells (128, 130), indicating an important aspect of population dynamics in IC blockade-mediated reversal of T cell exhaustion. A similar population of CD8 T cells responding to IC blockade, (PD1^int^/TCF-1^+^), has been recently described as precursors of terminally exhausted cells (PD1^high^/TCF-1^−^/TIM-3^+^) to be distinguished from memory precursors cells (PD1^−^/TCF-1^+^) on the basis of several epigenetic and transcriptional alterations such as higher expression of CXCR5 and Slamf6 (199).

By using a combined experimental and computational approach, Singer et al. (105) described mutually exclusive gene modules to distinguish dysfunctional from activated T cells in a murine colon carcinoma model. In particular, metallothionins, responsible for regulating the intracellular zinc metabolism, and zinc-dependent TFs were found to be highly enriched in dysfunctional CD8 TILs. GATA-3, a zinc-finger TF, consistently emerged as a driver of T cell dysfunction. Moreover, the expression of the co-inhibitory receptors PD-1 and TIM-3 was maintained upon metallothion-deletion, being uncoupled from the gene dysfunctional module (105).

Epigenetic studies also helped in identifying patterns distinguishing T cell exhaustion from T cell activation/memory profile. Recent epigenetic studies in mice and humans indicate that exhausted T cells represent a unique T cell lineage, compared to effector and memory T cells and are a stable, distinct and disease-relevant cell type (109, 111, 112).

HIV-1- and HCV-specific CD8 T cell genomes present a high accessibility to exhaustion-associated nucleotide regions. On the opposite, the genome of CMV-specific CD8 T cells is characterized by a higher accessibility to memory-specific nucleotide regions (109). Interestingly, the accessibility to exhaustion-specific regions is reduced in CD8 T cells specific for HCV epitopes that undergo viral escape (109), indicating that chronic exposure is needed to shape exhaustion-associated epigenetic imprinting.

Studies focusing on *Pdcd1* locus revealed that during the effector phase of an acute LCMV infection, the promoter regions were largely demethylated to become remethylated as the infection solved and CD8 T cell memory formed (106, 287). In the context of a chronic LCMV infection, the demethylation observed in the *Pdcd1* locus during chronic LCMV infection was instead stable and no remethylation was observed, even when viral titers and PD-1 protein expression by exhausted CD8 T cells decreased (106) or after transfer in recipient mice (260). Along the same lines, the unmethylated state of the *Pdcd1* locus did not change in T cells from subjects with a viral load controlled by ART for several years or from elite controllers (107). This suggests that the epigenetic program of the PD-1 locus is stabilized after prolonged exposure to HIV-1 virus despite different levels of PD-1 surface expression. Consistently, the transcriptome and the epigenome of terminally exhausted CD8 T cells (PD-1^high^/TCF-1^−^/TIM-3^+^) are stably rewired and resistant to remodeling after PD-1 blockade (111, 114) (Figure 2).

These data strongly suggest that epigenetic remodeling may be required to further improve strength and breadth of the efficacy of immune checkpoint blockade.

In conclusion, exhausted T cells can be distinguished from activated T cells by the higher and sustained co-expression of IC molecules, as well as by a phenotype skewed toward effector memory cells with reduced co-stimulatory molecules expression. Moreover, the systemically induced immune activation and the stable transcriptional and epigenetic imprinting established during T cell exhaustion do not allow the restoration of IC molecules expression to the levels measured in healthy donors even after antigen removal/reduction. Among the TFs analyzed, T-bet, and EOMES allow the distinction between activated and exhausted CD8 T cells in HIV-1 infection, while metallothionins and GATA-3 have been suggested as discriminators in cancer patients.

## Conclusions

Recent major advances in immunotherapy ultimately demonstrated the potentiality of the immune system in disease control. However, they also proved that existing strategies are hampered by the immune tolerance established by IC expression on T cells. In addition, despite the significant difference in the availability of clinical information concerning immunotherapy efficacy in cancer and HIV-1 infection, there is still a long way to go for the scientific community to decipher the mechanisms of immunosuppression in different indications. Recently, new technological advances (such as mass cytometry, single cell sequencing, ATAseq, metabolomics) are allowing a deeper examination of the molecular properties of dysfunctional T cells at the single cell level. These studies represent milestones for the comprehension of T cell biology in the context of complex TME, dominated by a high heterogeneity of cellular subsets and in HIV-1 infection where current immunotherapy may not improve T cell responses (64, 115). These data may lead to the understanding of new specific features of disease evolution and drive novel immunotherapeutic approaches.

## Author Contributions

SV and SB wrote the manuscript. All authors contributed to the article and approved the submitted version.

## Conflict of Interest

The authors declare that the research was conducted in the absence of any commercial or financial relationships that could be construed as a potential conflict of interest.
